# Supplementary material for: Improved implementation of aspirin in pregnancy among Dutch gynecologists: Surveys in 2016 and 2021
Source: PLoS One. 2022 Jun 9;17(6):e0268673. doi: 10.1371/journal.pone.0268673 (PMC9182337; doi:10.1371/journal.pone.0268673)
Supplement: S1 Table — HDP, hypertensive disorders of pregnancy; PE, preeclampsia; HELLP, hemolysis elevated liver enzymes low platelets; GA, gestational age; PIH, pregnancy induced hypertension; FGR, fetal growth restriction; SPTB, spontaneous preterm birth; SLE, systemic lupus erythematosus; APS, antiphospholipid syndrome; BMI, body mass index. (DOCX) [file pone.0268673.s001.docx]

| **GENERAL QUESTIONS** | | |
| --- | --- | --- |
| *Question* | *Answer* | *Nature of question* |
| How many years do you work in obstetrics? | a. <5 years  b. 5-15 years  c. >15 years | Multiple choice, one answer possible |
| In which type of hospital do you work at the moment? | a. University  b. Teaching  c. Non-teaching | Multiple choice, one answer possible |
| Are you registered as a consultant obstetrician? | a. Yes  b. No | Multiple choice, one answer possible |
| How many half-days a week do you perform obstetric care? | a. None or only when on duty  b. 1-4 half-days  c. 5 or more half-days | Multiple choice, one answer possible |
| **INDICATIONS FOR ASPIRIN** | | |
| *Question* | *Answer* | *Nature of question* |
| **History of recurrent miscarriages**  *Do you prescribe aspirin for …* | | |
| Recurrent miscarriages | a. Yes  b. No | Multiple choice, one answer possible |
| If yes, do you start aspirin preconceptional? | a. Yes  b. No | Multiple choice, one answer possible |
| **Hypertensive disorders of pregnancy (HDP)**  *Do you prescribe aspirin for …* | | |
| PE/HELLP with delivery <34 weeks of GA | a. Yes  b. No | Multiple choice, one answer possible |
| PE/HELLP with delivery between 34-37 weeks of GA | a. Yes  b. No | Multiple choice, one answer possible |
| PE/HELLP with delivery ≥37 weeks GA | a. Yes  b. No | Multiple choice, one answer possible |
| PIH | a. Yes  b. No | Multiple choice, one answer possible |
| **History of fetal growth restriction (FGR)**  *Do you prescribe aspirin for …* | | |
| FGR with delivery <34 weeks of GA | a. Yes  b. No | Multiple choice, one answer possible |
| FGR with delivery between 34-37 weeks of GA | a. Yes  b. No | Multiple choice, one answer possible |
| FGR with delivery ≥37 weeks GA | a. Yes  b. No | Multiple choice, one answer possible |
| **Spontaneous preterm birth** |  |  |
| *Do you prescribe aspirin for …* |  |  |
| Spontaneous preterm birth <34 weeks of GA | a. Yes  b. No | Multiple choice, one answer possible |
| **Auto-immune diseases**  *Do you prescribe aspirin for …* | | |
| SLE | a. Yes  b. No | Multiple choice, one answer possible |
| APS | a. Yes  b. No | Multiple choice, one answer possible |
| Other auto-immune disease | a. Yes  b. No | Multiple choice, one answer possible |
| If yes, for which other auto-immune disease? |  | Open question |
| **Maternal illnesses**  *Do you prescribe aspirin for …* | | |
| Chronic hypertension | a. Yes  b. No | Multiple choice, one answer possible |
| Diabetes mellitus type I or II | a. Yes  b. No | Multiple choice, one answer possible |
| Kidney disease | a. Yes  b. No | Multiple choice, one answer possible |
| **Moderate risk factors**  *Do you prescribe aspirin for …* | | |
| Nulliparity | a. Yes  b. No | Multiple choice, one answer possible |
| Advanced maternal age ≥40 years | a. Yes  b. No | Multiple choice, one answer possible |
| Pregnancy interval >10 years | a. Yes  b. No | Multiple choice, one answer possible |
| BMI >35kg/m^2^ | a. Yes  b. No | Multiple choice, one answer possible |
| Family history of PE/HELLP | a. Yes  b. No | Multiple choice, one answer possible |
| Multiple gestation | a. Yes  b. No | Multiple choice, one answer possible |
| Oocyte donation | a. Yes  b. No | Multiple choice, one answer possible |
| Combination of ≥2 of mild risk factors | a. Yes  b. No | Multiple choice, one answer possible |
| **Other**  *Do you prescribe aspirin for …* | | |
| Other indications | a. Yes  b. No | Multiple choice, one answer possible |
| If yes, for which indications? Please specify. |  | Open question |
| **PRESCRIPTION OF ASPIRIN** |  |  |
| *Question* | *Answer* | *Nature of question* |
| Which dose of aspirin do you prescribe? | … mg/day | Open question |
| Which time of administering aspirin do you advice? | a. In the evening  b. In the morning  c. At a fixed time during the day | Multiple choice, one answer possible |
| At what GA do you advice to start aspirin? | … weeks of GA | Open question |
| At what GA do you advice to stop aspirin? | … weeks of GA | Open question |
| *Do you advice to stop aspirin in case of …* |  |  |
| Vaginal blood loss | a. Yes  b. No | Multiple choice, one answer possible |
| Signs of preterm birth | a. Yes  b. No | Multiple choice, one answer possible |
| Stomach complaints | a. Yes  b. No | Multiple choice, one answer possible |
| Other reason | a. Yes  b. No | Multiple choice, one answer possible |
| If yes, for which reason? Please specify. |  | Open question |
