# Supplementary figures and images for: Improved implementation of aspirin in pregnancy among Dutch gynecologists: Surveys in 2016 and 2021
Source: PLoS One. 2022 Jun 9;17(6):e0268673. doi: 10.1371/journal.pone.0268673 (PMC9182337; doi:10.1371/journal.pone.0268673)

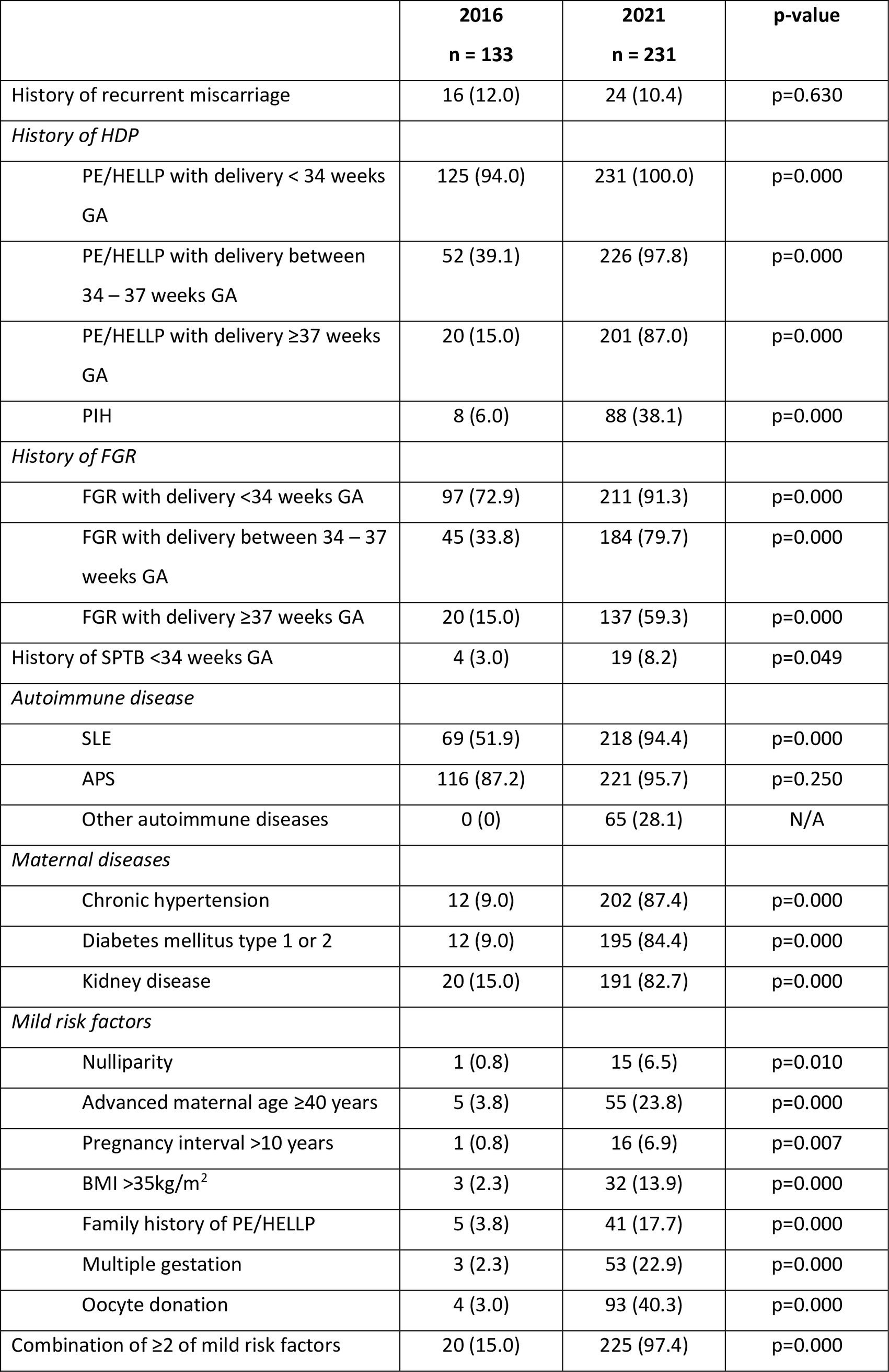

Supplement: S2 Table — Data are depicted as number (%). HDP, hypertensive disorders of pregnancy; PE, preeclampsia; HELLP, hemolysis elevated liver enzymes low platelets; GA, gestational age; PIH, pregnancy induced hypertension; FGR, fetal growth restriction; SPTB, spontaneous preterm birth; SLE, systemic lupus erythematosus; APS, antiphospholipid syndrome; BMI, body mass index; N/A, not available. (TIF) [file pone.0268673.s002.tif]
