# Supplementary material for: Improved implementation of aspirin in pregnancy among Dutch gynecologists: Surveys in 2016 and 2021
Source: PLoS One. 2022 Jun 9;17(6):e0268673. doi: 10.1371/journal.pone.0268673 (PMC9182337; doi:10.1371/journal.pone.0268673)
Supplement: Attachment — Submitted filename: Rebuttal letter.docx [file pone.0268673.s003.docx]

Academic Editor dr. S. Ornaghi
c/o PLOS One

Amsterdam, 4^th^ of April 2022

Dear dr. S. Ornaghi,

With the helpful suggestions from the editor and reviewers, we were able to improve our manuscript entitled ***“Improved implementation of aspirin in pregnancy among Dutch gynaecologists: surveys in 2016 and 2021”***, which we would like to resubmit for publication in PLOS One. Below you will find a point-by-point reply to the comments.

We look forward to your editorial decision.

Yours sincerely,

On behalf of all authors,

Jeske M. bij de Weg, MD

Amsterdam UMC location VUmc
Department of Obstetrics and Gynaecology
De Boelelaan 1117, 1081 HV, Amsterdam, the Netherlands
j.bijdeweg@amsterdamumc.nl

**Point-by-point reply to the comments of the editor and reviewers**

Manuscript Number: PONE-D-22-03768

In the point-by-point reply, revised text is presented between quotation marks. In the revised manuscript, all the changes are marked by tracked changes.

Reviewer #1:

I found this research on the improved implementation of aspirin in pregnancy among Dutch gynecologists interesting. The study deals with a highly topical obstetric issue yet to be defined at international level and shows the practical difficulties in implementing a guideline. The data are clearly presented and adequately support the conclusions and limitations. The major criticism is that adherence to the research involved a low percentage of gynecologists 13.6% in 2016 and 15.3% in 2021.

*Answer: We would like to thank the reviewer for the critical evaluation and compliments. We agree on the comment concerning the low response rate and added to the limitation section of the discussion on page 11, line 252-253: “A limitation of our study is the relatively low response rates of 13.6% and 15.3% in 2016 and 2021, respectively.”*

The recipients of the questionnaire is unclear in some parts of the manuscript - gynecologists (in training)???

*Answer: Due to this comment, we were able to further clarify our study population. We have explained in material and methods section page 4 lines 71-74 that in 2016 only gynecologist received the invitation for the survey, and in 2021 also the residents. Throughout the manuscript we replaced “gynecologists (in training)” by “gynecologists and residents”.*

Reviewer #2:

The author sought to perform a survey on the implementation of the use of aspirin in preventing preeclampsia after the introduction of national guidelines in the Netherlands comparing 2016 and 2020. This is a very nice example of National improvement of health practice. One limitation, well described by the authors, is the tertiary care background of those who participated. I would suggest to the authors to add in the “limitations” also the low rate of responders: around 13%, which is quite low.

*Answer: We thank the reviewer for their interest and comment. The same comment was made by reviewer #1. The low response rate is added to the limitation section of the discussion. See above answer for details.*

The authors presented a detailed description of the change in timing of the administration (evening versus other timings) and in the prescription of the dosage (low such as 80-100 mg versus high dosage as described in the ASPRE trial 150 mg).

I suggest to the Authors to perform in the next years a comparison between those gynecologists who rely on the anamnestic screening of preeclampsia (as stated by the Dutch National guidelines) compared to the bio markers+ultrasound+history based screening proposed by the FMF and used in the ASPRE trial. I suggest to include this topic in the Conclusions as future research.

*Answer: We want to thank the reviewer for this suggestion. We agree with the reviewer that optimizing screening for preeclampsia should be performed in future research in order to improve preventive therapy. We will keep in mind and discuss in our research group the suggestion made for future research. In the future section of the discussion on page 10, lines 231-233, we added the following: “The Fetal Medicine Foundation provides a biomarkers, ultrasound and history based prediction tool to identify women at high-risk for preterm PE.(47) Such a prediction method was tested in the ASPRE trial, detecting 76% of the preterm PE cases.(34)”. In the conclusion, page 11, line 264, we stated: “… screening tools for obstetrical complications and improvement of …”.*

Editorial remarks:

Please provide additional details regarding participant consent. In the ethics statement in the Methods and online submission information, please ensure that you have specified (1) whether consent was informed and (2) what type you obtained (for instance, written or verbal, and if verbal, how it was documented and witnessed). If your study included minors, state whether you obtained consent from parents or guardians. If the need for consent was waived by the ethics committee, please include this information.

*Answer: Since the survey was anonymous, informed consent is not required and therefore not obtained. The following was added to the online ethics statement: “No informed consent was obtained, since for an anonymous survey it is not required.”*

Please include your full ethics statement in the ‘Methods’ section of your manuscript file. In your statement, please include the full name of the IRB or ethics committee who approved or waived your study, as well as whether or not you obtained informed written or verbal consent. If consent was waived for your study, please include this information in your statement as well.

*Answer: The ethics statement made in the online submission process was added to the manuscript on page 4, lines 77-79: “The study was approved by the medical ethical committee of the Amsterdam UMC location VUmc (2020.0712). No informed consent was obtained, since for an anonymous survey it is not required.”*

Please upload a new copy of Figure 1 as the detail is not clear.

*Answer: A new file of Figure 1 is uploaded.*
